# Comparison of clinical features and outcomes of staphylococcus aureus vertebral osteomyelitis caused by methicillin-resistant and methicillin-sensitive strains

**DOI:** 10.1186/2193-1801-2-283

**Published:** 2013-06-27

**Authors:** Shinichi Inoue, Tokuhide Moriyama, Yutaka Horinouchi, Toshiya Tachibana, Fumiaki Okada, Keishi Maruo, Shinichi Yoshiya

**Affiliations:** Departments of Orthopaedic Surgery, Hyogo College of Medicine, 1-1 Mukogawa-cho, Nishinomiya, Hyogo, 663-8501 Japan

**Keywords:** Spondylitis, Vertebral osteomyelitis, Methicillin-resistant S. aureus (MRSA), Staphylococcus aureus, Vancomycin

## Abstract

The causative organism of vertebral osteomyelitis (VO) was almost exclusively *Staphylococcus aureus*. The purpose of this study was to delineate the differences in clinical features and outcomes between patients with methicillin-resistant Staphylococcus aureus (MRSA) and methicillin-sensitive Staphylococcus aureus (MSSA) VO. This study retrospectively reviewed 85 consecutive patients with VO treated between 2005 and 2011. Surgical site infections were excluded. Diagnosis was made by cultures of either blood or biopsied samples. We identified 16 cases of MRSA VO and 14 cases of MSSA VO. The average follow-up period was 18.5 months. Clinical features and outcomes were analyzed. Males were more likely to have MRSA VO than MSSA VO (87.5% vs. 35.7%). In regards to the number of co-morbidities, patients with MRSA VO had significantly more co-mobidities than patients with MSSA VO. Additionally, the rate of patients who underwent surgical procedure (excluding spinal surgeries in the affected region) within 3 months were significantly higher in the MRSA VO group than the MSSA VO group (56.3% vs. 14.3%). White blood cell counts and C-reactive protein levels in patients with both strains significantly improved 4 weeks after the initial treatment compared with the pretreatment values. The recurrence rate within 6 months tended to be higher for MRSA VO (37.5% vs. 7.1%), but no significant difference in mortality was observed between the two VO types. In conclusion, male sex, multiple co-morbidities and previous non-spine surgery were significant risk factors for VO due to MRSA as compared to MSSA. The recurrence rate within 6 months tended to be higher for MRSA VO. Patients with MRSA VO should be monitored carefully for recurrence by sequential clinical, radiographic, and laboratory examinations during the treatment course.

## Introduction

The number of patients with pyogenic vertebral osteomyelitis (VO) has increased steadily in recent years (Zimmerli 2010; Mylona et al. 2009; Cebrián Parra et al. 2012). The incidence of VO has been estimated to be 2.4 cases per 100,000 people and increases with increasing age (from 0.3 per 100,000 among people aged < 20 years to 6.5 per 100,000 among people aged >70 years) (Zimmerli 2010; Cebrián Parra et al. 2012). In 1931, Hatch (1931) reviewed the literature and reported that the causative organism was almost exclusively Staphylococcus aureus (SA). After initiation of antibiotic use, Sapico and Montgomerie (1980) reported in 1979 that 67% bacteria that caused spinal infections were the gram-positive type, and 55% were methicillin-sensitive Staphylococcus aureus (MSSA). SA remains a common pathogen and a major cause of morbidity and mortality (Zimmerli 2010; Mylona et al. 2009; Cebrián Parra et al. 2012; Yoon et al. 2010; Corrah et al. 2011; Mete et al. 2012).

The first strains of methicillin-resistant Staphylococcus aureus (MRSA) was reported in 1961 (Barber 1961). Thereafter, the number of adult patients with MRSA bacteremia increased with time and has been a growing problem. Previous studies have demonstrated a significant increase in mortality among patients who had MRSA bacteremia compared with mortality among patients who had MSSA bacteremia (Cosgrove et al. 2003; Shurland et al. 2007). In regards to other infectious diseases (meningitis (Chang et al. 2001), endocarditis (Yoon et al. 2005), arthritis (Al-Nammari et al. 2007a), liver abscess (Ferreira et al. 2011), and spinal epidural abscess (Huang et al. 2012)), there have been recent studies comparing the clinical characteristics of infection caused by MSSA and MRSA strains.

Recently, several authors have reported clinical characteristics of MRSA spondylitis (Al-Nammari et al. 2007b; Livorsi et al. 2008; Priest & Peacock 2005). However, few studies have compared the clinical features of spondylitis due to MSSA and MRSA. The purpose of the present study was to delineate the differences in clinical features and outcomes between patients with MRSA VO and MSSA VO.

## Materials and methods

This study design was approved by the review board of our institute. We retrospectively reviewed 85 consecutive patients who had pyogenic spondylitis treated between January 2005 and December 2011 at Hyogo College of Medicine Hospital. Our facility is a 910-bed tertiary care university hospital in Japan that provides medical care to approximately 300,000 inpatients, including over 8000 surgical operations, and services 600,000 outpatients per year. Our hospital has 34 highly specialized medical treatment departments plus an emergency department, and it has 26 medical treatment centers/facilities, including a cancer center. On the basis of the study by Al-Nammari et al. (2007b), we used the following inclusion criteria in this study:

-  No previous local spinal surgery at any time
-  Compatible clinical history
-  Compatible imaging
-  MSSA or MRSA isolated from bone, intervertebral disc, or paravertebral biopsies and/or MRSA or MSSA isolated from blood cultures in patients with a clinical and radiological history of spondylodiscitis
-  Follow-up ≥ 6 months
-  Case notes, image, and electronic patient record available

A BacT-ALERT 3D (Sysmex-biomerieux, Tokyo, Japan) was used for blood cultures. The Auto-Scan W/A and Comb Panel (Siemens) and standard techniques were used to identify isolates and determine susceptibility. Microdilution using the methods of Clinical Laboratory Standards Institute were used to determine methicillin susceptibility.

Thirty consecutive patients with SA VO met the inclusion criteria (MSSA, 14 cases; MRSA 16, cases). The patients’ demographic data, clinical characteristics, and radiographic and laboratory features were recorded and compared between the MSSA and MRSA strains. The patient demographic data collected included age, gender, height, weight, body mass index (BMI), and comorbidities. Co-morbidities included heart disease, respiratory disease, liver disease, urinary tract infection, hemodialysis, diabetes mellitus, steroids use, malignancy, chemotherapy, and postsurgical states. Use of the phrase “post-surgical states” was meant for patient who underwent any invasive surgeries within 3 months, excluding surgical site infection after spinal surgery. Among clinical characteristics, pain due to infection, fever (> 38°C), neurological deficit, and septic shock and the onset of symptoms (classification of (Kulowski 1936)) were reviewed. Radiographic features, including the number, localization, and staging (classification of (Griffiths & Jones 1971)) of the vertebral body infections, were investigated using X-rays, and the presence of abscess was investigated by magnetic resonance imaging (MRI). Laboratory features were evaluated by obtaining white blood cell (WBC) counts, C-reactive protein (CRP) levels, and serum albumin (Alb) levels. Clinical outcomes included antimicrobial duration, length of hospital stay, surgical intervention, relapse and mortality within 6 months, and laboratory findings (WBC counts and CRP levels) at the 4th week after antibiotic treatment. Surgical treatment is required if conservative treatments are ineffective, if there is concomitant neurological deficit, or if there is development or worsening of deformity due to severe destruction of the anterior column. Relapse of infection was defined as developed symptoms related to recurrence of inflammation markers (WBC counts and CRP levels) with or without worsening of radiographic findings. Patient deaths were classified as either that caused by infection or by the underlying disease.

### Statistical analysis

All numerical results were presented as means ± SDs. The unpaired *t*-test and Fisher’s exact probability test were used to compare differences between the groups. The paired *t*-test was used to compare continuous variables during the clinical course. P values < 0.05 were considered to indicate statistical significance. Statistical analysis was performed using SPSS Statistics 20.0 (IBM, IL, USA).

## Results

A positive microbiological diagnosis was made for 49 (57.6%) patients with VO (Table 1). The most frequently identified organism was SA (30 patients, 61.2%). The mean age and gender ratio (male/female) were 65.6 ± 13.4 years (range, 14–88 years) and 19/11, respectively. The average follow-up was 18.5 ± 18.4 months (range, 2–62 months). Five patients died within 6 months after initial therapy.

**Table 1:** **Causative organisms identified in the 49 cases of vertebral osteomyelitis**

Organism	Number of cases (%)
*Staphylococcus aureus*	30/49 cases (61.2%)
*Coagulase-Negative Staphylococci*	10/49 cases (20.4%)
*Streptococcus sp*	3/49 cases (6.1%)
*Fungus*	2/49 cases (4.1%)
*Escherichia coli*	1/49 case (2.0%)
*Serratia marcescens*	1/49 case (2.0%)
*Citrobacter sp*	1/49 case (2.0%)
*Stenotrophomonas maltophilia*	1/49 case (2.0%)

Patient demographics and clinical characteristics (Table 2).

**Table 2:** **Patient demographics and clinical characteristics**

	MSSA-VO (14)	MRSA-VO (16)	***P***-value
Patient demographics			
Age	65.4 ± 17.2	65.9 ± 9.5	0.918
Male	37.5% (5)	87.5% (14)	0.007*
Height	155.8 ± 9.5	164.3 ± 7.6	0.010*
Weight	54.4 ± 8.2	56.0 ± 11.2	0.655
BMI	22.4 ± 2.9	20.6 ± 3.5	0.154
Mean number of co-morbidities	1.2 ± 0.8	2.2 ± 1.1	0.011*
Diabetes mellitus	21.4% (3)	37.5% (6)	0.338
Heart disease	14.3% (2)	6.3% (1)	0.586
Respiratory disease	7.1% (1)	6.3% (1)	0.922
Liver disease	21.4% (3)	6.3% (1)	0.315
Urinary tract infection	7.1% (1)	6.3% (1)	0.922
Hemodialysis	7.1% (1)	6.3% (1)	0.922
Malignancy	14.3% (2)	37.5% (6)	0.226
Use steroids	14.3% (2)	18.8% (3)	0.743
Chemotherapy	0% (0)	18.8% (3)	0.228
Post-surgical states	14.3% (2)	56.3% (9)	0.026*
Clinical characteristics			
Pain	100% (14)	93.8% (15)	0.467
Fever (>38°C)	64.3% (9)	50% (8)	0.484
Neurological deficit	50% (7)	50% (8)	1.000
Septic shock	21.4% (3)	43.8% (7)	0.260
Kulowski classification: acute type	57.1% (8)	62.5% (10)	0.284
Number of vertebral body infected	2.3 ± 0.8	2.2 ± 0.7	0.719
Localization of vertebral body infected			
Cervical	35.7% (5)	18.8% (3)	
Thoracic	28.6% (4)	37.5% (6)	
Lumber	35.7% (5)	43.8% (7)	0.721
X-ray: Griffiths classification			
(Destructive and osteosclerotic stage)	78.6% (11)	87.5% (14)	0.642
MRI: presence of abscess	85.7% (12)	62.5% (10)	0.226
WBC mean (x 10^3^/ul)	12.6 ± 6.0	12.2 ± 5.4	0.847
CRP mean (mg/dl)	15.4 ± 9.3	15.8 ± 12.9	0.919
Albumin mean (g/dl)	2.7 ± 0.6	3.0 ± 0.5	0.217

*Statistically significant.

*BMI* indicates body mass index, *WBC* white blood cell, *CRP* C-reactive protein.

Fourteen patients had MSSA VO and 16 had MRSA VO. There was no statistical difference in patient age (65.4 ± 17.2 vs. 65.9 ± 9.5, P = 0.918) between MSSA VO and MRSA VO. There were significantly more males in the MRSA VO group than in the MSSA VO group (87.5% vs. 35.7%, *P* = 0.007). Height was significantly higher in MRSA VO; however, there were no significant differences in weight and BMI between the groups. The mean number of co-morbidities for each patient in the MRSA VO group was significantly larger than that in the MSSA VO group (2.2 ± 1.1 vs. 1.2 ± 0.8, *P* = 0.011). There was no significant difference in the prevalence of each of the co-morbidities between the MSSA and MRSA patients. History of preceding invasive surgical procedures (postsurgical status) within 3 months except for spinal surgery in the corresponding region was confirmed significantly more often in the MRSA VO group than the MSSA VO group (56.3% vs. 14.3%, P = 0.026). Seven patients with MRSA VO and 2 patients with MSSA VO had undergone mainly gastroenterological and cardiovascular surgeries, whereas the remaining 3 patients with MRSA VO had undergone orthopedic surgery for mainly community-acquired infectious arthritis at a previous hospital. Among the MRSA VO patients, 89% with post-surgical states were diagnosed as having pyogenic spondylodiscitis within 1 month from the past operation.No statistically significant differences between the MSSA VO and MRSA VO groups were observed with regard to pain, fever, neurological deficit, and the onset of symptoms. In the radiographic images, the number, localization, and staging of the vertebral body infections as well as the presence of abscess were similar between the MRSA and MSSA groups. In the MSSA VO and MRSA VO groups, the mean WBC counts, CRP levels, and Alb levels were 12.6 ± 6.0 vs. 12.2 ± 5.4 × 103/μl, 15.4 ± 9.3 vs. 15.8 ± 12.9 mg/dl, and 2.7 ± 0.6 vs. 3.0 ± 0.5 g/dl, respectively; there were no significant differences between the 2 groups (*P* = 0.847, *P* = 0.919, *P* = 0.217, respectively).

Clinical outcomes (Table 3).

**Table 3:** **Clinical outcomes**

	MSSA-VO (14)	MRSA-VO (16)	***P***-value
Duration of antimicrobial therapy (days)	95.4 ± 76.0	74.7 ± 42.4	0.362
Length of hospital stay (days)	103.0 ± 83.5	103.1 ± 76.0	0.998
Surgical intervention	64.3% (9)	37.5% (6)	0.272
Relapse within 6 months	7.1% (1)	37.5% (6)	0.086
Mortality within 6 months	21.4% (3)	12.5% (2)	0.642
WBC post therapy at 4th week (× 10^3^/ul)	6.3 ± 2.1	8.6 ± 3.4	0.047*
CRP post therapy at 4th week (mg/dl)	3.1 ± 2.6	2.8 ± 2.6	0.733

*Statistically significant.

*WBC* indicates white blood cell, *CRP* C-reactive protein.

There were no significant differences in the antimicrobial duration and length of hospital stay between the MSSA and MRSA groups. The operation intervention rates for MSSA and MRSA were 64.3% and 37.5%, respectively, which were not significantly different (*P* = 0.272). Mainly Reasons for surgical were neurological deficit in 66.7% of MRSA VO and 77.8% of MSSA VO. In approaches of operation, anterior approach alone were employed for 5 cases with MRSA VO and 7 case with MSSA VO, while posterior approach with or without anterior debridement were adopted for 2 cases with MRSA VO and 2 cases with MSSA VO. Blood examination 4 weeks after the initial treatment showed that the WBC counts in the MRSA VO group tended to be higher than those in the MSSA VO group. There was no apparent difference in the CRP levels between the groups. In addition, after treatment, the WBC counts and CRP levels in both groups improved significantly compared with the pretreatment values. The recurrence rate within 6 months tended to be higher in the MRSA VO group (37.5% vs. 7.1%, *P* = 0.086), whereas no significant difference in mortality was observed between the groups (12.5% vs. 21.4%, *P* = 0.642). In the 16 patients with MRSA VO, primary antibiotic therapy consisted of linezolid in 9 patients (56.3%, mean 25 days), vancomycin in 5 patients (31.3%, mean 40 days), teicoplanin in 1 patient (6.3%, 28 days), and daptomycin in 1patinets (6.3%, 42 days). Incidences of recurrence in patients had undergone treatment with vancomycin and linezolid were 60% (3 patients) and 33.3% (3 patients), respectively.

## Discussion

As previously reported in other studies (Zimmerli 2010; Mylona et al. 2009; Cebrián Parra et al. 2012; Hatch 1931; Sapico & Montgomerie 1980; Yoon et al. 2010; Corrah et al. 2011; Mete et al. 2012), SA was the most common cause of hematogenous VO (61.2%) in our series. Moreover, MRSA VO occurred in larger number of patients (16) than MSSA VO (14 patients) in this study population. Livors et al. (2008) reported that MRSA was responsible for 57% vertebral SA infections and MSSA for 43%. In this study, there was a significantly greater male:female ratio in patients with MRSA VO than in those with MSSA VO. Based on the results of the nation-wide survey for 857 MRSA isolate, Yanagihara indicated male predominance for this organism accounting for 66.3% of the total population (Yanagihara et al. 2012). (Al-Nammari et al. 2007b) reported that 85% of patients with MRSA spondylitis were male. The reasons for these gender differences are not known.

In our series, patients with MRSA VO had significantly more co-morbidities compared to patients with MSSA VO (2.2 vs. 1.2). Moreover, 73% patients in this study had 2 or more co-morbidities. (Cebrián Parra et al. 2012) reported that 1 or more comorbid diseases were present in 73 (68%) of 108 patients with infectious discitis. (Al-Nammari et al. 2007b) demonstrated that multiple underlying diseases were present in 76% patients who had hematogenous MRSA spondylodiscitis. In previous studies that investigated the type of infectious disease, such as bacteremia (Shurland et al. 2007), endocarditis (Yoon et al. 2005), and arthritis (Al-Nammari et al. 2007a), MRSA-infected patients had more co-morbidities than did MSSA-infected patients. The risk for MRSA carriage may be higher because multiple underlying diseases would lead to more opportunities for contact with medical institutions.

The present study showed the preceding surgical procedure was a factor associated with occurrence of MRSA VO. History of surgical procedures such as gastroenterological surgery and cardiovascular surgery within 3 months, was confirmed in 66.7% patients. (Ferreira et al. 2011) reported that MRSA liver abscess was the most important complication associated with post-abdominal surgery. The remaining 3 patients with related orthopedic surgery were previously diagnosed as having MRSA spondylitis after orthopedic surgery for infectious MRSA at a different institution. Interestingly, most patients (89%) having post-surgical states had indications of MRSA VO within 1 month after previous surgery. Al-Nammari et al. (2007b) reported that all patients with MRSA spondylodiscitis had undergone invasive medical procedures in the preceding 6 months. We believe that the reasons for MRSA spondylodiscitis were close contact with medical institutions, intravenous catheter use, previous antibacterial therapy, compensatory anti-inflammatory response syndrome, or preceding MRSA bacteremia.

The mortality rate within 6 months was 16.7% (5 cases) in our series. Previous studies have shown that the mortality rates for spondylitis were 6%–38% (Mylona et al. 2009; Hatch 1931; Sapico & Montgomerie 1980; Mete et al. 2012; Al-Nammari et al. 2007b; Livorsi et al. 2008; Priest & Peacock 2005) and that the survival rate for MRSA spondylitis was worse than that for spondylitis due to other pathogens. In the analysis of the treatment course, no significant difference was observed in mortality within 6 months between the MSSA VO and MRSA VO groups. (Cosgrove et al. 2003), in a comparison with MSSA bacteremia, reported that MRSA bacteremia was associated with a significantly higher mortality rate. However, a recent study demonstrated no significant difference in mortality between MRSA and MSSA bacteremia (Wang et al. 2008).

MRSA spondylitis tends to recur after temporary remission. In SA spondylitis including MRSA-infected patients, recent studies have demonstrated that the relapse rate was 14%–17% (Livorsi et al. 2008; Priest & Peacock 2005). (Al-Nammari et al. 2007b) showed that 29% survivors at 1 year had MRSA bacteremia and spondylitis recurrence. The reason for the high recurrence may be that vancomycin was less effective for osteomyelitis caused by MRSA because the levels and bactericidal activity achieved in bone may have been low (Gelfand & Cleveland 2004; Aspinall et al. 1995). Most patients with MRSA VO who relapsed had mainly undergone treatment with vancomycin. (Gelfand & Cleveland 2004) suggested that monotherapy with vancomycin for treatment of MRSA bacteremia, while apparently successful by conventional clinical and laboratory criteria, may not prevent the development of VO. (Aspinall et al. 1995) recommended that combination antibiotic therapy with vancomycin and rifampin with or without low-dose gentamicin might be useful for MRSA spondylitis. Previous study reported that linezolid was superior to vancomycin in the treatment MRSA infections disease, however, linezolid can cause mild, reversible, time-dependent myelosuppression, such as thrombocytopenia, particularly when patients are treated for >14 days (Kohno et al. 2007). The guideline in 2011 (Liu et al. 2011) prepared by the Infectious Diseases Society of America recommended that antibiotics available for parenteral administration include vancomycin and daptomycin, with or without rifampin. Although the adequate length for intravenous antimicrobial therapy is unknown, a minimum 8-week course is recommended (Liu et al. 2011). Therefore, patients with MRSA-VO should be carefully monitored for clinical, radiographic, and laboratory features during and after the initial therapy, because vancomycin monotherapy may not prevent the progression of osteomyelitis due to MRSA.

There were several limitations in this study. First, our study was a single-institution retrospective clinical review, and our small sample size did not provide enough power to detect a difference between clinical features of MSSA VO and MRSA VO. Moreover, the use of a multivariate analysis may not be fully validated to identify independent factors predicting MRSA VO in this study population. With an average follow-up of 18 months, we could not examine quality of life after treatment in this study population. Secondly, not all of the patients underwent examinations to detect SA carriage by nasal mucosa cultures. (von Eiff et al. 2001) reported that in patients with SA bacteremia, there was a strong correlation between strains that colonized nasal tissues and those isolated from,the blood of the patients. Accordingly, in the present study, it was impossible to differentiate between hematogenous SA spondylitis and the carriage of bacteria in the nasal mucosa as a cause of infection.

## Conclusions

Our study demonstrated that MRSA spondylitis was associated with male predominance and multiple co-morbidities. Post-surgical states within 3 months were the most important underlying condition of patients with MRSA spondylitis. We supposed that the risk of MRSA carriage might be higher because multiple underlying diseases would lead to more opportunities for contact with medical institutions. The recurrence rate within 6 months tended to be higher in the patients with MRSA VO, but no significant difference in mortality was observed between the groups. Based on these study results, we propose that hematogenous SA spondylitis should be considered as the initial tentative diagnosis to start antimicrobial administration for patients with VO, and then patients with MRSA VO should be carefully monitored for recurrence by sequential clinical, radiographic, and laboratory examinations during the treatment course.

## References

[CR1] Al-Nammari SS, Bobak P, Venkatesh R (2007a). Methicillin resistant Staphylococcus aureus versus methicillin sensitive Staphylococcus aureus adult haematogenous septic arthritis. Arch Orthop Trauma Surg.

[CR2] Al-Nammari SS, Lucas JD, Lam KS (2007b). Hematogenous methicillin-resistant Staphylococcus aureus spondylodiscitis. Spine.

[CR3] Aspinall SL, Friedland DM, Yu VL, Rihs JD, Muder RR (1995). Recurrent methicillin-resistant Staphylococcus aureus osteomyelitis: combination antibiotic therapy with evaluation by serum bactericidal titers. Ann Pharmacother.

[CR4] Barber M (1961). Methicillin-resistant staphylococci. J Clin Pathol.

[CR5] Cebrián Parra JL, Saez-Arenillas Martín A, Urda Martínez-Aedo AL, Soler Ivañez I, Agreda E, Lopez-Duran Stern L (2012). Management of infectious discitis. Outcome in one hundred and eight patients in a university hospital. Int Orthop.

[CR6] Chang WN, Lu CH, Wu JJ, Chang HW, Tsai YC, Chen FT (2001). Staphylococcus aureus meningitis in adults: a clinical comparison of infections caused by methicillin-resistant and methicillin-sensitive strains. Infection.

[CR7] Corrah TW, Enoch DA, Aliyu SH, Lever AM (2011). Bacteraemia and subsequent vertebral osteomyelitis: a retrospective review of 125 patients. QJM.

[CR8] Cosgrove SE, Sakoulas G, Perencevich EN, Schwaber MJ, Karchmer AW, Carmeli Y (2003). Comparison of mortality associated with methicillin-resistant and methicillin-susceptible Staphylococcus aureus bacteremia: a meta-analysis. Clin Infect Dis.

[CR9] Ferreira JP, Abreu MA, Rodrigues P, Carvalho L, Correia JA (2011). Meticilin resistant Staphylococcus aureus and liver abscess: a retrospective analysis of 117 patients. Acta Med Port.

[CR10] Gelfand MS, Cleveland KO (2004). Vancomycin therapy and the progression of methicillin-resistant Staphylococcus aureus vertebral osteomyelitis. South Med J.

[CR11] Griffiths HE, Jones DM (1971). Pyogenic infection of the spine. A review of twenty-eight cases. J Bone Joint Surg Br.

[CR12] Hatch ES (1931). Acute osteomyelitis of the spine: Report of case with recovery. Review of the literature. New Orleans Med Surg J.

[CR13] Huang PY, Chen SF, Chang WN, Lu CH, Chuang YC, Tsai NW (2012). Spinal epidural abscess in adults caused by Staphylococcus aureus: clinical characteristics and prognostic factors. Clin Neurol Neurosurg.

[CR14] Kohno S, Yamaguchi K, Aikawa N, Sumiyama Y, Odagiri S, Aoki N (2007). Linezolid versus vancomycin for the treatment of infections caused by methicillin-resistant Staphylococcus aureus in Japan. J Antimicrob Chemother.

[CR15] Kulowski J (1936). Pyogenic osteomyelitis of the spine. J Bone Joint Surg.

[CR16] Liu C, Bayer A, Cosgrove SE, Daum RS, Fridkin SK, Gorwitz RJ (2011). Clinical practice guidelines by the infectious diseases society of america for the treatment of methicillin-resistant Staphylococcus aureus infections in adults and children. Clin Infect Dis.

[CR17] Livorsi DJ, Daver NG, Atmar RL, Shelburne SA, White AC, Musher DM (2008). Outcomes of treatment for hematogenous Staphylococcus aureus vertebral osteomyelitis in the MRSA ERA. J Infect.

[CR18] Mete B, Kurt C, Yilmaz MH, Ertan G, Ozaras R, Mert A (2012). Vertebral osteomyelitis: eight years' experience of 100 cases. Rheumatol Int.

[CR19] Mylona E, Samarkos M, Kakalou E, Fanourgiakis P, Skoutelis A (2009). Pyogenic vertebral osteomyelitis: a systematic review of clinical characteristics. Semin Arthritis Rheum.

[CR20] Priest DH, Peacock JE (2005). Hematogenous vertebral osteomyelitis due to Staphylococcus aureus in the adult: clinical features and therapeutic outcomes. South Med J.

[CR21] Sapico FL, Montgomerie JZ (1980). Vertebral osteomyelitis in intravenous drug abusers: report of three cases and review of the literature. Rev Infect Dis.

[CR22] Shurland S, Zhan M, Bradham DD, Roghmann MC (2007). Comparison of mortality risk associated with bacteremia due to methicillin-resistant and methicillin-susceptible Staphylococcus aureus. Infect Contr Hosp Epidemiol.

[CR23] von Eiff C, Becker K, Machka K, Stammer H, Peters G (2001). Nasal carriage as a source of Staphylococcus aureus bacteremia. Study Group. N Engl J Med.

[CR24] Wang JL, Chen SY, Wang JT, Wu GH, Chiang WC, Hsueh PR (2008). Comparison of both clinical features and mortality risk associated with bacteremia due to community-acquired methicillin-resistant Staphylococcus aureus and methicillin-susceptible S. aureus. Clin Infect Dis.

[CR25] Yanagihara K, Araki N, Watanabe S, Kinebuchi T, Kaku M, Maesaki S (2012). Antimicrobial susceptibility and molecular characteristics of 857 methicillin-resistant Staphylococcus aureus isolates from 16 medical centers in Japan (2008-2009): nationwide survey of community-acquired and nosocomial MRSA. Diagn Microbiol Infect Dis.

[CR26] Yoon HJ, Choi JY, Kim CO, Kim JM, Song YG (2005). A comparison of clinical features and mortality among methicillin-resistant and methicillin-sensitive strains of Staphylococcus aureus endocarditis. Yonsei Med J.

[CR27] Yoon SH, Chung SK, Kim KJ, Kim HJ, Jin YJ, Kim HB (2010). Pyogenic vertebral osteomyelitis: identification of microorganism and laboratory markers used to predict clinical outcome. Eur Spine J.

[CR28] Zimmerli W (2010). Clinical practice. Vertebral osteomyelitis. N Engl J Med.

